# Work-Related Musculoskeletal Disorders Risk Assessment during Manual Lymphatic Drainage with Compressive Bands among Physiotherapists

**DOI:** 10.3390/healthcare12010118

**Published:** 2024-01-04

**Authors:** Julien Jacquier-Bret, Philippe Gorce

**Affiliations:** 1 International Institute of Biomechanics and Occupational Ergonomics, 83418 Hyères, France; gorce@univ-tln.fr; 2 Université de Toulon, CS60584, 83041 Toulon, France; 3 Hôpital Léon Bérard, Avenue du Docteur Marcel Armanet, 83418 Hyères, France

**Keywords:** occupational health, posture, massage, RULA, REBA, risk, ergonomic assessment

## Abstract

Background: Complete decongestive therapy is the standard treatment for lymphedema. Manual lymphatic drainage and short-stretch multilayer compression bandaging are two daily stages of complete decongestive therapy during which physiotherapists work with patients. Objective: The aim of this study was to assess the risks of musculoskeletal disorders to which physiotherapists are exposed during these two phases. Method: Five physiotherapists performed five 20 min manual lymphatic drainages, followed by the compression bandaging phase. From the video recordings, 8477 postures defined by 13 joint angles were grouped into clusters using hierarchical cluster analysis. The risk of musculoskeletal disorders in physiotherapists’ postures was assessed using ergonomic tools. Results: Seven clusters, called generic postures (GP), were identified and defined throughout the mean joint angle values and standard deviation. Four seated GPs were found for the drainage phase, and three standing GPs were identified for the bandaging phase. This phase corresponded to a quarter of the total duration. The GP’s ergonomic scores ranged from 4.51 to 5.63 and from 5.08 to 7.12, respectively, for the Rapid Upper Limb Assessment (RULA) and Rapid Entire Body Assessment (REBA). GP1, GP3, and GP4 presented the highest ergonomic scores (RULA scores: 5.27 to 5.63; REBA scores: 6.25 to 7.12). The most affected areas were the neck (flexion > 20° for all GPs), trunk (flexion between 25 and 30° for GP2, and GP7 during the bandaging phase and GP4 during the drainage phase), and shoulder (flexion and abduction >20° for all GPs except GP5). Conclusions: These results highlighted that the two complete decongestive therapy phases could be described as a combination of GP. Ergonomic assessment showed that compression bandaging as well as drainage phases expose physiotherapists to moderate musculoskeletal disorder risks that require “further investigation and change soon”.

## 1. Introduction

In their professional practice, physiotherapists (PTs) perform various activities such as manual therapy techniques, therapeutic exercises, transferring or lifting patients or moving heavy equipment (with or without assistance), education, making clinical assessments, writing patient case notes and reports, and collecting statistics, etc. [1,2]. Some of these activities are physically demanding, often performed in awkward postures, and repeated many times a day throughout the year. Under these conditions, the occurrence of musculoskeletal disorders (MSDs) is frequent, as reported in studies of physiotherapists in different countries [3,4]. Based on surveys, authors have estimated the overall 12-month prevalence of MSDs at over 80% [5,6]. Others have studied the MDS prevalence by body area through cross-sectional studies [7,8] and literature reviews [9]. The most frequently reported affected areas were the neck, lower back, shoulder, and wrist/hand. The prevalence reported for these areas varies widely from one study to another. The prevalence of MSDs ranged from 16% [10] to 66.5% [11] for the neck, from 19.4% [12] to 66% [6] for the lower back, from 14.8% [1] to 62.2% [11] for the shoulder, and from 21.8% [5] to 46% [8] for the wrist/hand.

Some studies have examined the main risk factors, the factors that could exacerbate symptoms, and the solutions and treatments implemented by physiotherapists to reduce the risk of MSDs and continue their practice. Many answers were directly related to awkward postures such as “bending or twisting” [13,14], “working in the same position for a long time” [1,15], “working in an awkward/cramped position” [13,14], and working near joint limits [1,16]. In the responses implemented to reduce the impact of MSDs during their practice, physiotherapists often reported changes in their working posture or in the way they positioned their patients [5,14]. All these results show that physiotherapists are exposed to MSDs.

Studies have focused on particular activities performed by physiotherapists, such as massage, transfers, and segmental mobilization. Massage is an important part of their work in some hospital centers. As a result, physiotherapists can be exposed to MSD risks, as demonstrated by Yoopat et al. [17] and Głowiński et al. [18]. Some massages have specific features, such as manual lymphatic drainage (MLD), which is used as a treatment technique for lymphedema (LE). LE is caused by a decrease in lymphatic transport capacity and/or an increase in lymphatic load. It is characterized by soft tissue swelling induced by the accumulation of fluid in extracellular spaces. It is a chronic condition that causes an increase in the size and weight of the affected areas, leading to impaired joint mobility and movement. It can induce postural alterations and pain that impair activities of daily living [19]. MLD is part of a larger procedure called complete decongestive therapy (CDT), currently recognized as the standard of care in the treatment of LE [20]. In addition to MLD, CDT incorporates (1) short-stretch multilayer compression bandaging (CB), (2) exercises to improve lymphatic pumping, (3) meticulous skin care of affected areas, and (4) the fitting of appropriate compression garments to maintain the reductions achieved by treatment [21]. The aim of CDT is to reduce your swelling, improve the condition of your skin, increase your mobility, reduce the risk of infection, and optimize your overall health. This practice involves awkward postures, particularly during the massage phase [22]. The issue associated with the study of MSD risks from long-term activity is the large number of postures adopted. One way of solving this problem is to group similar postures into clusters, for example, with hierarchical cluster analysis [23]. Jacquier-Bret et al. have used this principle and introduced the notion of generic posture (GP) [22]. This approach enables a complex activity to be described by a reduced number of postures defined by mean angular values and their standard deviation. The MSD risks associated with the use of these GPs have been assessed using ergonomic tools such as Rapid Upper Limb Assessment (RULA [24]) or Rapid Entire Body Assessment (REBA [25]) [26]. However, in these studies, the authors focused only on the MLD phase. However, the CB phase is relevant to analyze since it is systematic, includes the application of several bands, is repeated several times a day, and represents a non-negligible amount of time during the MLD. To our knowledge, no study has investigated the MSD risk analysis of this phase through the postures observed among physiotherapists in the context of a CDT.

The aim of this study was therefore to analyze the risks of MSD associated with the practice of CDT using ergonomic assessment tools, taking into account both stages: MLD and CB. Posture analysis was conducted using the GP concept introduced by Jacquier-Bret et al. [22]. The underlying question is whether CB is a risk phase in the apparition of MSDs.

## 2. Materials and Methods

### 2.1. Participants

One male and four female right-handed physiotherapists (40.2 ± 11.3 years old, 166.2 ± 6.9 cm, 63.0 ± 7.6 kg, and BMI: 22.9 ± 3.6, 17.6 ± 9.8 years of experience) were included in the analysis of two phases of the CDT. They worked full-time (35 h spread over 5 days) in the neurology department of the Léon Bérard hospital. None of them suffered from musculoskeletal disorders or pathologies that could affect their care practices (evaluation done with the Nordic Musculoskeletal Questionnaire [27]). The entire protocol was presented to the physiotherapists, and everyone gave their written consent before the beginning of the experience. The protocol was in agreement with the Helsinki agreement [28] and was approved by the ethics committee of the Léon Bérard hospital (LBCE-2023-31).

### 2.2. Experimental Design

**Figure 1:**
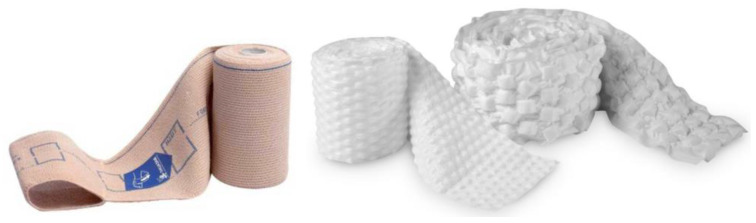
Compression bandaging wrapped around the limb of a patient with lymphedema. On the left is an elastic compression band. On the right is compression device is composed of foam blocks encased between two non-woven bandages.

Each physiotherapist performed the first two stages of CDT 5 times: (1) a 20 min MDL using the Leduc technique with an intensity of 40 mmHg and (2) multilayer, short-stretch compression bandaging (CB) on the affected limb. The CB phase was performed as follows: an elastic band protects the skin from irritation and allergies (Figure 1, left panel). A second band creates a drainage action thanks to foam cubes (Figure 1, right panel). A third compressive band holds the device in place, providing contention and rigidity.

Two numeric cameras (Samsung Galaxy S20, 60 Hz, Samsung Electronics, Seoul, Republic of Korea) were used to film each physiotherapist’s massage directly in their department according to their time schedule and the patient’s admission. The two cameras were positioned at 90° to each other to continuously film the physiotherapists in the frontal and sagittal planes without any inconvenience [22].

### 2.3. Data Analysis–Posture Definition

From these video recordings, the physiotherapists’ postures observed during MLD and CB phases of the CDT were defined through thirteen joint angles by two experts every 5 s, i.e., 8477 postures: neck, trunk, shoulders, elbows, hips, and knees flexion/extension and abduction/adduction or inclination. All joint angles were defined following the recommendations of the International Society of Biomechanics [29,30]. A model developed with Matlab (The Mathworks, Inc., Natick, MA, USA) was used to compare the postures estimated by the experts and those obtained from the video. Joint angle values were adjusted if necessary to define the final posture. Flexions/extensions correspond to movements in the sagittal plane around the mediolateral axis. Abduction/adduction and inclination correspond to movements in the frontal plane around the anteroposterior axis of the body. Neck and trunk rotations were estimated using the model.

### 2.4. Data Analysis–Posture Clustering

A hierarchical cluster analysis (HCA) was performed on the matrix 8477 postures × 13 joint angles using Matlab (Statistical Toolbox, The Mathworks, Inc., Natick, MA, USA). The clustering procedure began by computing the sum of squares of the error between each element using the Ward linkage method [31]. The two closest are then paired into binary clusters. Ward’s method aims to choose the successive clustering steps in such a way as to minimize the increase in the sum of squares of the error at each step. This procedure was iterated continuously to generate a clustering tree that was represented as a dendrogram (showing the linking distance between each cluster). All postures attached to the same branch are considered close and form an independent cluster of postures. The agglomeration coefficient was used to quantitatively identify the clusters, and their consistency was verified by a visual inspection of the dendrogram.

From all the postures included in a cluster, an average posture was computed (13 average joint angles ± standard deviation) and was called generic posture (GP). The frequency of use of each GP throughout the two CDT phases was also computed.

### 2.5. Data Analysis–Ergonomic Assessment of Posture

The risk of musculoskeletal disorders was assessed using the RULA and REBA. The RULA was chosen because it is the most widely used tool and focuses on the upper limbs, which are mainly involved in massage [32]. REBA was added because it correlates well with RULA [33] and includes the lower limbs involved in the CB phase.

The joint angle values were used to inform the posture scores for the upper limb (group A for RULA and group B for REBA) and neck/trunk/leg (group A for REBA and group B for RULA). From these scores and the method’s abacuses, the final RULA and REBA scores were obtained for each posture (Table C in RULA and REBA grids). These scores reflect the level of MSD risk associated with posture. The RULA method reported 4 levels on a 7 point scale: level 1, score 1–2: “negligible risk, no action needed if not maintained or repeated for long periods” (green color); level 2, score 3–4: “low risk, further investigation is needed, and changes may be required” (yellow color); level 3, score 5–6: “medium risk, investigation, and changes are required soon” (orange color); level 4, score 6+: “high risk, investigation, and changes are required immediately” (red color). The REBA method uses 5 levels on a 12 point scale: level 1, score 1: “negligible risk” (green color); level 2, score 2–3: “low risk, change may be needed” (yellow color); level 3, score 4–7: “medium risk, further investigate, change soon” (orange color); level 4, score 8–10: “high risk, investigate, and implement change” (red color); level 5, score 11+: “very high risk, implement change” (dark red color).

First, RULA and REBA scores were computed for each of the 8744 postures using the joint angle values to complete the ergonomic RULA and REBA grids. Then, mean RULA and REBA scores were computed for each cluster to assess the MSD risk associated with each GP.

Second, the MSD risk was assessed for each physiotherapist in two different ways. On one hand, the time spent at each risk level was calculated for the MLD phase, the CB phase, and the both phases together. The mean RULA and REBA scores were then computed from the scores of each posture (Equations (1) and (2)). On the other hand, the mean RULA and REBA scores were computed from the frequency and the mean scores relative to each GP (Equations (3) and (4)).

(1) $$Mean\;RULA\;score\;from\;all\;posutre=\frac{\sum_{i=1}^{n}RULA\;score}{n}$$

(2) $$Mean\;REBA\;score\;from\;all\;posutre=\frac{\sum_{i=1}^{n}REBA\;score}{n}$$

(3) $$Mean\;RULA\;score\;from\;GPs=\sum_{i=1}^{7}{GP}_{i}\;RULA\;score\times {GP}_{i}\;frequency$$

(4) $$Mean\;REBA\;score\;from\;GPs=\sum_{i=1}^{7}{GP}_{i}\;REBA\;score\times {GP}_{i}\;frequency$$

with n corresponding to the number of postures included in a phase.

### 2.6. Statistical Analysis

The 13 joint angles and RULA/REBA scores were dependent variables. Values for each GP were presented as the mean value (±standard deviation). Due to the non-normality of the data, the non-parametric Kruskal–Wallis test was used to compare the dependent variables for each GP using Statistica software (Statistica 7.1, Statsoft, Tulsa, OK, USA). The significance level was set at 5%.

## 3. Results

The mean duration of two phases of the CDT was 28.26 ± 4.02 min, with 21.99 ± 3.51 min for the MLD phase and 6.27 ± 0.80 min for the CB phase.

### 3.1. Generic Posture Definition with the Hierarchical Cluster Analysis

**Figure 2:**
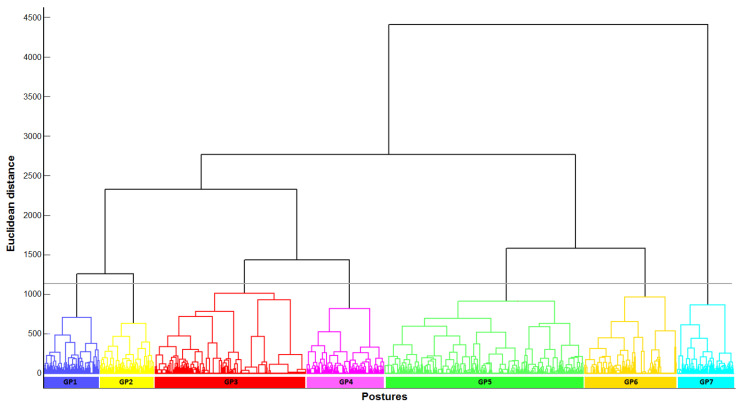
Ward’s minimum variance linkage dendrogram representing the seven-cluster solution of the hierarchical cluster analysis achieved from all measured postures during manual lymphatic drainage. The horizontal black line represents the cluster separation threshold. Each color represents a cluster of similar postures called “generic posture”.

The HCA evidenced 7 clusters of postures based on the 13 joint angles defined for the 8477 postures considered (Figure 2). Each cluster was represented by a GP defined by the mean values and standard deviation of the 13 joint angles (Table 1). GP frequency varied from less than 10% (GP1, GP2, and GP7) to 28.98% (GP5).

All GPs had significant neck flexion (>20°, highest RULA and REBA local scores). GP3 and GP5 had statistically the lowest flexion (20.92 ± 7.33° and 20.10 ± 11.48°, respectively, *p* < 0.05), and GP1 and GP2 presented the highest flexion (26.74 ± 9.07° and 27.38 ± 9.50°, respectively). Inclination and rotation values were low (around 5° or less). Inclination was significantly highest for GP3 (7.51 ± 7.66°) and GP6 (6.05 ± 9.09°, *p* < 0.05), while rotation was the most important for GP5 (5.44 ± 8.56°, *p* < 0.05). Trunk flexion was significantly lowest for GP5 (15.05 ± 7.56°) and GP6 (13.52 ± 9.36°) and highest for GP1 (30.32 ± 10.46°) and GP7 (29.27 ± 12.70°, *p* < 0.05). Inclination was maximal for GP4 (17.21 ± 11.04°) and GP6 (10.38 ± 8.72°), while rotation (>10°) was the highest for GP5 (10.62 ± 10.77°) compared with the other GPs (*p* < 0.05). Large differences were observed for shoulder angles. Flexion and abduction ranged from low values (flexion: 13.81 ± 12.67° and abduction: 13.03 ± 9.93° for GP5) to values greater than or equal to 50° (flexion: 51.63 ± 15.54° for GP3; abduction: 49.50 ± 18.90° for GP6). A high joint range was also found for the elbow, with flexion values comprising between 38.63 ± 13.71° (GP1) and 86.65 ± 14.51° (GP5). For the lower limbs, three major positions were evidenced: a standing GP (GP7 with angles close to 0°), two standing positions with one leg resting on the massage table (GP1 and GP2) characterized by hip flexion of 75° and knee flexion > 130°, and four seated positions (GP3, GP4, GP5, and GP6), including one with significant hip abduction (27.04°, GP4, *p* < 0.05).

### 3.2. Ergonomic Assessment of Posture during CDT

As shown in Table 2, the risk of MSDs related to the two phases of the CDT was assessed as low (four GPs with scores above 4.5) to medium (three GPs with scores above 5) according to RULA, and medium (all GPs with scores above 5) regarding REBA. Scores ranged from 4.51 to 5.63 and from 5.08 to 7.12, respectively, for RULA and REBA. GP5 presented the lowest ergonomic scores (RULA: 4.51 and REBA: 5.08) while the highest were found for GP4 (RULA: 5.63 and REBA: 7.12).

### 3.3. GP Distribution per CDT Phase and Physiotherapist

Table 3 shows the distribution of GPs for the MLD phase, the CB phase, and the two phases combined per physiotherapist. The results showed that the three standing GPs (GP1, GP2, and GP7) were preferentially used during the CB phase. The other seated GPs (GP3 to GP6) were used for the MLD phase. For both the MLD and CB phases, the ergonomic scores reflect a medium risk of MSD (mean RULA: 5.02 and mean REBA: 5.82) for all CDTs. The distribution of GPs by phase showed the postural preferences of each physiotherapist. PT1 mainly used GP3 and GP5 for the MLD phase and GP7 for the CB phase. PT2 massages preferentially with GP5 and uses GP5 for the CB phase. PT3 uses GP3, GP5, and GP6 for the MLD phase and mainly GP2 for the CB phase. PT4 uses GP5 for the MLD phase and GP1 and GP2 for the CB phase. Finally, PT5 uses GP4 for the MLD phase and GP1 and GP7 for the CB phase.

**Figure 3:**
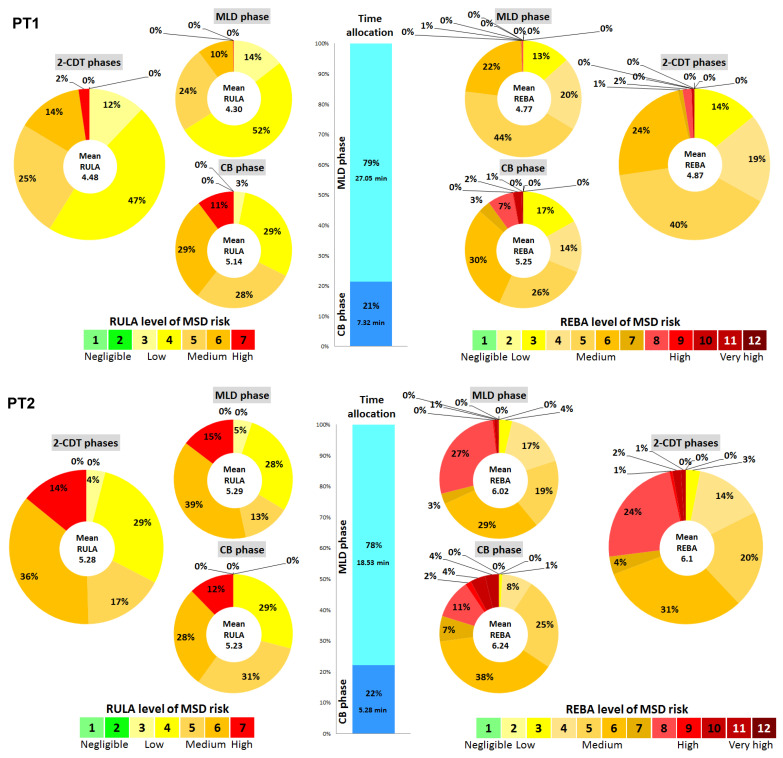

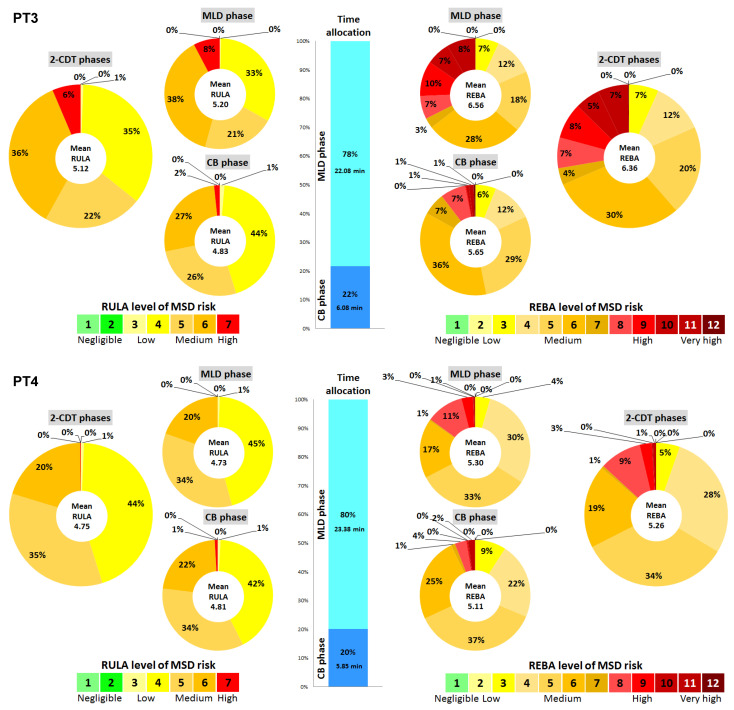

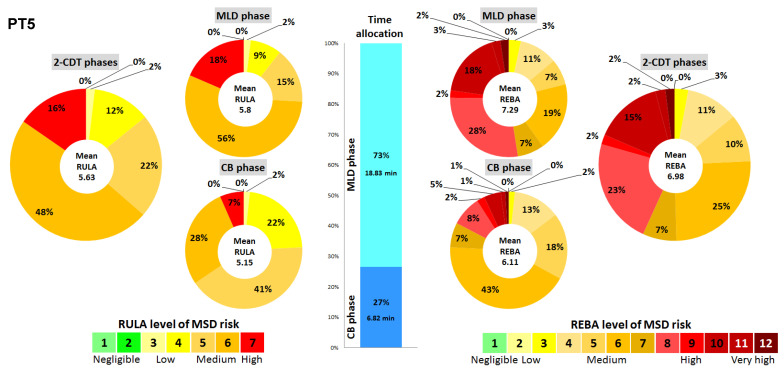
Distribution of MSD risk by physiotherapist using RULA (left panel) and REBA (right panel) scores for the MLD phase, the CB phase, and the 2-CDT phases. The central histogram represents the relative proportion of MLD and CB phases. Mean RULA and REBA scores were computed from the scores obtained for all postures used by each physiotherapist (PT).

Figure 3 illustrates the distribution of MSD risk by physiotherapist using RULA (left panel) and REBA (right panel) scores for the MLD phase, the CB phase, and the two CDT phases combined. In contrast to Table 2, mean RULA and REBA scores were computed from the scores obtained for all postures used by each physiotherapist. Results showed that MLD and CB phases presented equivalent risks across all PTs. RULA scores ranged from 4.48 to 5.63 for the two CDT phases combined, from 4.30 to 5.8 for the MLD phase, and from 4.81 to 5.23 for the CB phase. The trend was identical for REBA: 4.87 to 6.98 for the full MLD, 4.77 to 7.29 for the MLD phase, and 5.11 to 6.24 for the CB phase. Across all two CDT phases, we found that for the highest level of MSD risk, corresponding to a high level of risk, a significant proportion of the MLD duration was present. For RULA, 6.6% to 8.2% of the time was spent with scores of 7, respectively, for the CB and MLD phases. For REBA, the proportion of high risk levels was higher: 12.6% to 21.6% for these two phases.

Finally, analysis of the two CDT phases showed that the CB phase accounted for between 20% and 27% of the total time for all PTs.

## 4. Discussion

The aim of this study was to quantify the risk of MSDs in physiotherapists when treating lymphedemas with complete decongestive therapy (CDT). The studied phases were manual lymphatic drainage (MLD) and short-stretch multilayer compression bandaging (CB). In the neurology department of the Léon Bérard hospital, physiotherapists perform CDT every day. They care for patients twice a day: in the morning, they perform the MLD and CB, and in the afternoon, they repeat the CB after the pressotherapy session. It therefore appears that the CB stage is a recurrent and repetitive activity for physiotherapists, especially when several bands are superimposed. However, this phase has never been taken into account in the assessment of MSD risks associated with MLD activities. Yet the results showed that this phase accounted for a quarter of the mean duration of MLDs, that is, an average of 6.27 min repeated twice a day.

The hierarchical cluster analysis methodology employed enabled the 8744 postures studied to be classified into 7 clusters known as generic postures (GP) [22]. As indicated by Jacquier-Bret et al., GP allow complex and long-duration tasks to be described through a limited number of key postures [22]. Despite a larger number of postures than in the previous work by Gorce et al. [26], conducted only on the MDL phase (8744 vs. 6594 postures) and an equivalent agglomeration coefficient of 1000, an identical number of GPs were highlighted and appeared distinctly in the dendrogram (Figure 2). Analysis of the mean joint angles defining each GP (Table 1) revealed that three GP corresponded to standing postures (GP1, GP2, and GP7) and four GP to sitting postures (GP3, GP4, GP5, and GP6). Two of the standing GPs (GP1 and GP2) were characterized by a leg resting on the massage table (significant hip and knee flexion). The third GP (GP7) corresponds to the standing posture. These three GPs were used preferentially during the CB phase (Table 3). To the best of our knowledge, no study has proposed specific postures to describe this activity. The four seated GPs were more commonly used for the MLD phase and can be found in previous work. Gorce et al. reported seven distinct GPs only for the MLD phase [26]. Four of these were found in the present study. GP3, GP4, GP5, and GP6 presented in Table 1 correspond to GP5, GP6, GP1, and GP3 in their study. This result shows that even though the MLD were performed at different times over a different duration, the same posture clusters and therefore the same GPs were found as the key postures in the execution of repetitive massage activities. As a result, the GPs presented in this work better describe the postures adopted during the CDT since they have been defined from both the MLD and CB phases. The MSD risk assessment associated with postures observed during CDT showed that, like the MLD phase, the CB phase presented significant levels of risk. The analysis revealed ergonomic RULA scores between 4.87 and 5.56 and REBA scores between 5.22 and 6.72. These scores correspond to a low (GP2 and GP7) to medium (GP1) level of risk for RULA [24] and to medium risk for the three GPs according to REBA [25]. However, the results showed that physiotherapists spend a significant part of the CB phase in postures with high ergonomic scores (RULA equal to 7 and REBA greater than or equal to 8), exposing them to a high risk of MSD. This result is reinforced by the fact that the relative proportion of the CB phase is important (i.e., one-fourth of the total duration) and that this activity is repeated several times a day (two to four patients per day with two applications per day). A further exacerbating factor is that more than half of these activities are performed on the lower limbs, requiring the handling of heavy loads (i.e., 16% of body mass [34]). With regard to MLD, the MSD risk levels observed were similar to those observed in previous studies over longer durations (3 and 6 months). Scores ranged from 4.46 to 5.87 for RULA [23] and from 5.06 to 7.39 for REBA [26], representing a medium-risk level.

The detailed analysis described in the present study reinforces the prevalence studies currently available in the literature over one year [6] or a full career [1], which show that physiotherapists are professionals with a high level of exposure to MSDs. Quantifying joint angles through GPs highlights the causes of MSD prevalence, whereas the literature proposes analyses through questionnaires (e.g., the Nordic Musculoskeletal Questionnaire [27]), which report the presence of pain and MSDs. Table 1 highlighted significant neck flexion (>20°) for the entire CDT (MLD and CB phases), which explains the high reported prevalence for this area (>60% [8,11]). Significant trunk flexion (25–30°) was also observed for GP1, GP2, GP7 (relating to the CB phase), and GP4 (for the MLD phase), which was linked to a prevalence of over 50% [5,35]. GP4 also had a high degree of rotation, which increases the risk of MSD. For the shoulder, except for GP5 with values below 15°, all GPs had values between 20 and 60°, responsible for the prevalence of MSDs of at least 40% reported in cross-sectional studies [6,13]. For the elbow, GP1 and GP3 showed low flexion values (35–45°), indicating an extended arm posture at the origin of the reported MSDs, with a prevalence of around 20–30% [7,36]. Finally, the detailed analysis by the physiotherapist illustrated their “postural habits” that vary from one practitioner to another, as shown in Table 3. PT1 and PT3 used GP3 and GP5 for MLD, while PT2 and PT4 used GP5. As for PT5, his behavior is different since he mainly uses GP4. For the CB phase, PT4 and PT5 used GP1, PT1 and PT5 used GP7 and PT3, and PT4 used GP2. Finally, PT2 used GP5. Such “postural habits” have not been demonstrated in the literature. This result shows that, despite the different combinations of GPs used, the risk of MSDs in both the CB and MLD phases is significant. The approach using GP could be used to propose individualized recommendations as part of MSD prevention. General recommendations as follows could be addressed to prevent the apparition of MSDs in physiotherapists during CDT:

- Use foam supports to create support for the upper limbs to reduce muscular strain on the shoulders.
- Position yourself facing the area to be massaged to avoid movements in the frontal plane (abduction and inclination), which increase physical demands and consequently the risk of MSDs.
- Choose postures that are as far away from joint limits as possible.
- Adjust table and seat heights to avoid awkward postures (RULA ≥ 5 and REBA ≥ 4).

These recommendations are focused on the objectives of the study. More generally, massage is considered a low-load activity (<15% of maximal voluntary contraction for shoulder muscles) [17]. Despite these findings, previous studies have shown that massage practitioners have a high overall prevalence of MSDs (71.4% [36] and 88.9% [18]). The authors agree that, despite the low physical load, physiotherapists should pay attention to their posture in order to reduce the risk of pain and injury. [11]. The quantified ergonomic assessment of postures during MLD showed that the risk of MSDs associated with this activity was moderate to high, requiring changes in the physiotherapist’s practice or work environment [26]. The results of the present study extend this observation to “secondary tasks” such as multilayer compression bandaging following an MLD as part of a CDT for the management of lymphedema. Despite the low physical load, the physiotherapists adopted risky postures equivalent to those of MLDs, that is, likely to generate MSDs in the long term. However, these results cannot be generalized to all physiotherapists, as their practice is directly linked to their environment and working conditions. The type of patient, the level of training and expertise of physiotherapists, the department in which they work, the presence and use of ergonomic equipment, etc. are all factors that could affect posture and the risk of MSDs. The analysis of postures therefore appears relevant and essential to explaining the origins of MSDs among physiotherapists. However, other manual therapies need to be studied to get a more precise idea of potential MSD risks, taking into account working conditions in different departments, that is, differences in clinical practice.

### Limitations

The main limitation concerns the number of physiotherapists and CDTs studied. A larger number of physiotherapists and CDT would enable us to consider other factors that may influence the occurrence of MSDs, such as practitioners’ experience, age, gender, place of practice, working conditions, etc.

MSD risks have been assessed using ergonomic tools, in which the efforts made are only taken into account macroscopically. It would be relevant to study the impact of finger force exertion on the risk of wrist and hand MSDs among physiotherapists.

In addition, measurements were taken under real working conditions in the neurology department of the Léon Bérard hospital. This observational approach allowed for qualifying and quantifying the postures adopted daily by physiotherapists. However, it did not enable us to assess the effects of specific adjustments, such as table and stool height, made by physiotherapists, on the risk of MSDs. It would be interesting to take them into account in future work.

## 5. Conclusions

The aim of this study was to investigate the risk of MSD during CDT, including the MLD phase and the CB, which have never been considered in MSD risk assessment. CDT was described by seven clusters of postures defined by a mean posture called generic posture (GP). Four seated GPs were found for the MLD phase, and three standing GPs were identified for the CB phase. The latter corresponds to a quarter of the duration of the MDLs and presents the same level of MSD risk as the MLD phase, that is, a medium level of risk requiring further intervention and change soon. This approach has enabled us to better understand the impact of CDT over a long period of time in order to protect physiotherapists in their occupational activity.

## Figures and Tables

**Table 1:** Mean (standard deviation) joint angles and frequency for each GP throughout all massages.

	GP1		GP2		GP3		GP4		GP5		GP6		GP7	
	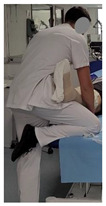		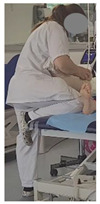		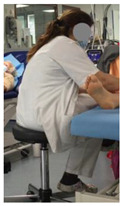		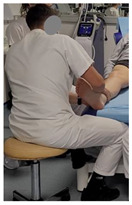		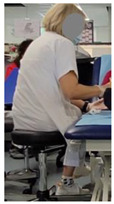		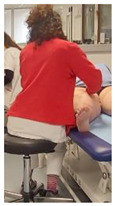		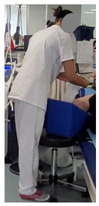	
Neck flexion (°)	26.74	(9.07) ^#343567^	27.38	(9.50) ^#343567^	20.92	(7.33) ^#12457^	23.27	(7.01) ^#12367^	20.10	(11.48) ^#12467^	23.06	(9.46) ^#12357^	25.82	(10.76) ^#123456^
Neck inclination (°)	5.52	(6.44) ^#234567^	3.99	(5.84) ^#1346^	5.33	(8.34) ^#1257^	7.51	(7.66) ^#1257^	2.94	(4.08) ^#13456^	6.05	(9.09) ^#1257^	3.63	(5.15) ^#1346^
Neck rotation (°)	2.82	(3.50) ^#23467^	3.03	(6.27) ^#15^	2.68	(4.55) ^#14567^	2.39	(2.84) ^#135^	5.44	(8.56) ^#23456^	3.04	(4.19) ^#135^	2.57	(3.78) ^#135^
Trunk flexion (°)	30.32	(10.46) ^#234567^	26.21	(11.86) ^#13567^	15.68	(8.99) ^#124567^	23.81	(7.39) ^#13567^	15.05	(7.56) ^#12347^	13.52	(9.36) ^#12347^	29.27	(12.70) ^#123456^
Trunk inclination (°)	7.42	(8.14) ^#34567^	7.96	(8.32) ^#467^	8.19	(7.99) ^#1467^	17.21	(11.04) ^#123567^	7.33	(6.67) ^#1467^	10.38	(8.72) ^#123457^	4.21	(5.54) ^#123456^
Trunk rotation (°)	4.67	(6.01) ^#56^	4.13	(6.05) ^#3456^	7.60	(8.45) ^#2567^	5.29	(5.56) ^#2567^	10.62	(10.77) ^#12347^	8.59	(9.03) ^#12347^	3.49	(4.86) ^#3456^
Shoulder flexion (°)	35.89	(16.96) ^#23567^	25.46	(15.24) ^#13457^	51.63	(15.54) ^#124567^	35.38	(11.86) ^#23567^	13.81	(12.67) ^#123467^	27.25	(13.87) ^#13457^	21.73	(15.11) ^#123456^
Shoulder abduction (°)	25.06	(13.53) ^#34567^	23.17	(15.06) ^#456^	23.48	(16.61) ^#14567^	30.14	(12.59) ^#123567^	13.03	(9.93) ^#123467^	49.50	(18.90) ^#123457^	20.42	(14.86) ^#13456^
Elbow flexion (°)	38.63	(13.71) ^#234567^	85.49	(8.75) ^#13467^	44.89	(16.53) ^#124567^	59.99	(20.05) ^#123567^	86.65	(14.51) ^#13467^	84.06	(16.33) ^#123457^	65.10	(25.95) ^#123456^
Hip flexion (°)	75.60	(17.76) ^#34567^	72.64	(15.85) ^#34567^	84.20	(8.17) ^#12457^	87.57	(6.65) ^#123567^	85.47	(7.02) ^#123467^	83.56	(6.50) ^#12457^	5.37	(15.16) ^#123456^
Hip abduction (°)	11.19	(13.75) ^#34567^	8.66	(12.79) ^#34567^	−2.84	(7.85) ^#12467^	27.04	(6.73) ^#123567^	−1.61	(12.99) ^#12467^	−4.41	(7.80) ^#123457^	0.00	(0.00) ^#123456^
Hip rotation (°)	0.00	(0.00)	0.00	(0.00)	0.00	(0.00)	0.47	(5.97)	0.00	(0.00)	0.00	(0.00)	0.00	(0.00)
Knee flexion (°)	134.60	(11.16) ^#34567^	133.57	(12.98) ^#34567^	84.27	(6.92) ^#12457^	90.07	(2.58) ^#123567^	88.70	(5.18) ^#123467^	83.61	(11.80) ^#12457^	0.18	(1.53) ^#123456^
Frequency (%)	8.13%		7.88%		22.11%		11.21%		28.98%		13.55%		8.14%	

GP1 to 7: generic posture 1 to 7. ^#X^: statistically different from GPX, with X between 1 and 7 for GP1 to GP7 (Kruskal–Wallis analysis, *p* < 0.05). Shoulder abduction: negative values correspond to adduction.

**Table 2:** Detailed and mean (standard deviation) RULA and REBA scores by GP computed from all postures included in each GP.

		GP1	GP2	GP3	GP4	GP5	GP6	GP7		
RULA score distribution by GP	1	-	-	-	-	-	-	-		
	2	-	-	-	-	-	-	-	RULA level of MSD risk	
	3	0.7%	1.9%	4.1%	3.4%	8.7%	0.3%	2.0%	1–2	Negligible risk. No action is needed
	4	10.9%	41.2%	27.6%	14.8%	46.1%	48.1%	35.4%	3–4	Low risk. Change may be needed
	5	32.9%	23.7%	20.3%	16.2%	31.3%	10.6%	38.7%	5–6	Medium risk. Further investigation. change soon
	6	42.2%	31.1%	33.6%	46.8%	13.8%	38.7%	21.4%	6+	High risk. Investigate and implement the change now
	7	13.2%	2.1%	14.4%	18.7%	0.1%	2.2%	2.5%		
Mean RULA score		5.56 (0.88) ^#23567^	4.90 (0.94) ^#1345^	5.27 (1.13) ^#124567^	5.63 (1.05) ^#23567^	4.51 (0.84) ^#123467^	4.94 (0.98) ^#1345^	4.87 (0.86) ^#1345^		
REBA score distribution by GP	1	-	-	-	-	-	-	-		
	2	-	-	-	-	-	-	-		
	3	1.7%	8.7%	-	0.3%	15.6%	5.4%	9.9%		
	4	2.8%	15.6%	3.7%	8.3%	30.2%	28.5%	18.0%	REBA level of MSD risk	
	5	15.4%	27.7%	38.5%	19.4%	19.6%	26.4%	31.9%	1	Negligible risk. No action is needed
	6	40.6%	30.5%	32.7%	18.3%	18.9%	17.0%	33.6%	2–3	Low risk. Change may be needed
	7	10.2%	0.9%	5.9%	3.7%	0.6%	1.5%	1.2%	4–7	Medium risk. Further investigation. change soon
	8	16.1%	12.7%	7.0%	28.8%	12.5%	7.1%	2.2%	8–10	High risk. Investigate and implement change
	9	1.7%	1.8%	2.3%	1.1%	2.0%	8.3%	1.2%	11+	Very high risk. Implement the change now
	10	8.6%	0.7%	5.9%	17.2%	0.1%	0.7%	1.2%		
	11	1.5%	1.2%	3.6%	1.9%	0.5%	5.1%	0.6%		
	12	1.5%	0.1%	0.4%	1.1%	0.0%	0.0%	0.4%		
Mean REBA score		6.72 (1.75) ^#23567^	5.57 (1.63) ^#13457^	6.25 (1.74) ^#124567^	7.12 (2.05) ^#23567^	5.08 (1.67) ^#123467^	5.69 (2.06) ^#1345^	5.22 (1.42) ^#12345^		

GP1 to 7: generic posture 1 to 7. ^#X^: statistically different from GPX, with X between 1 and 7 for GP1 to GP7 (Kruskal–Wallis analysis, *p* < 0.05).

**Table 3:** Distribution of GPs and mean RULA/REBA score for the MLD phase, the CB phase, and the two CDT phases for each physiotherapist.

		PT1			PT2			PT3			PT4			PT5			RULA	REBA
		MLD	CB	MLD + CB	MLD	CB	MLD + CB	MLD	CB	MLD + CB	MLD	CB	MLD + CB	MLD	CB	MLD + CB		
GP1	Freq. (%)	-	15.9%	3.4%	-	3.8%	0.8%	-	26.3%	5.7%	5.5%	33.3%	11.1%	13.4%	40.3%	20.6%	5.56	6.72
GP2	Freq. (%)	-	14.8%	3.2%	-	6.0%	1.3%	-	65.2%	14.1%	2.1%	41.6%	10.0%	7.7%	20.3%	11.0%	4.90	5.57
GP3	Freq. (%)	46.4%	-	36.5%	18.3%	13.2%	17.1%	38.6%	-	30.2%	17.2%	-	13.8%	10.9%	-	8.0%	5.27	6.25
GP4	Freq. (%)	2.9%	-	2.3%	10.9%	4.1%	9.4%	-	-	-	12.5%	-	10.0%	52.0%	1.0%	38.5%	5.63	7.12
GP5	Freq. (%)	34.3%	0.2%	27.1%	62.1%	25.9%	54.0%	26.3%	2.5%	21.2%	46.3%	4.3%	37.9%	9.2%	-	6.7%	4.51	5.08
GP6	Freq. (%)	16.4%	7.5%	14.5%	8.8%	15.8%	10.4%	33.8%	-	26.5%	12.2%	0.9%	9.9%	6.9%	0.5%	5.2%	4.94	5.69
GP7	Freq. (%)	-	61.5%	13.1%	-	31.2%	6.9%	1.3%	6.0%	2.3%	4.1%	19.9%	7.2%	-	37.9%	10.1%	4.87	5.22
Mean RULA score from GP		4.97	4.98	4.98	4.81	4.90	4.82	4.95	5.06	4.98	4.91	5.10	4.95	5.38	5.16	5.32		
Mean REBA score from GP		5.78	5.54	5.74	5.58	5.55	5.56	5.74	5.84	5.76	5.71	5.86	5.74	6.57	5.92	6.40		

MLD = manual lymphatic drainage; CB = short-stretch multilayer compression bandaging; PT = physiotherapist; GP = generic posture; RULA = rapid upper limb assessment; REBA = rapid entire body assessment.

## Data Availability

Data are available on request.
